# Progress in Obstetrics

**Published:** 1901-11-09

**Authors:** 


### Progress in Obstetrics.

**Puerperal Eclampsia.**—Dr. H. Oliphant Nicholson[1] calls attention to the relation which probably exists between eclampsia and the thyroid gland. He points out that the blood changes which follow thyroidectomy resemble those met with during pregnancy, viz., a diminution in the number of red corpuscles and albumin, and an increase in the number of white corpuscles, with a deficiency of the solid constituents of the serum. There are also during pregnancy certain toxins present in the blood, and although the nature and origin of these toxins is not clear, there is some reason to believe that the fœtus plays an important part in their production, because after its death the various symptoms usually abate. If the toxin is of fœtal origin it is unlikely that we can exert any control over its formation, so that our efforts in the avoidance of eclampsia must be directed towards the maintenance of efficient renal function, whereby the poison may be eliminated. A checking of this elimination by the kidney would result in the development of all the symptoms of eclampsia. Therefore the whole question of treatment, prophylactic and otherwise, turns upon elucidating the cause of this renal arrest, and Dr. Nicholson suggests that defective action of the thyroid gland may be the cause of this arrest of renal function. He points out that thyroid extract has a distinct diuretic effect in diseases of the kidney, although apparently negative in healthy persons, it also lowers the blood pressure and dilates the arteries. The internal secretion of the suprarenal bodies, on the other hand, acts in an antagonistic manner, and one might assume that a balance between the two resulted in a certain arterial tone. If the thyroid secretion should become diminished the suprarenal influence on the arterioles, including those of the kidney, would be very profound. As regards treatment he recommends that in the early stages when there is increased tension of the pulse, and diminution of urine, thyroid extract should be given twice or thrice daily, and proteid food forbidden. If convulsions have already occurred, then the use of thyroidin by the mouth would not be rapid enough. Liquor thyroidii, in from 10 to 15 minim doses, should be given by hypodermic injection and repeated every hour or two if not followed by signs of improvement. For the immediate treatment of the convulsion he recommends morphia, which should be given in large doses. This probably relieves the spasm of the arteries and diminishes blood pressure. Dr. Pollock Simpson[2] draws attention to an important point in the administration of morphia in these cases. He says we must examine the urine qualitatively as well as quantitatively, and should the paraglobulin be in greater proportion than the serum-albumin, it belongs to the class of cases when altered blood pressure rather than nephritis is the cause of the albuminuria, and the former are the cases likely to be benefited by the administration of morphia. Dr. Jardine[3] believes that the toxin which produces eclampsia is probably a waste product from tissue metabolism, but that its exact source of origin is still doubtful. From the degenerative changes that have been found in the liver in many cases he thinks that this organ plays an important part in ridding the system of the poison. Dr. Jardine also points out that the poison seems to act on the fœtus in much the same way as it does on the mother. He has seen cases in which the child suffered from convulsions during the first few days of its life, and he has also found albumin in the urine of several children whose mothers had severe albuminuria, but who were rescued from eclampsia by prompt treatment. He strongly advocates the treatment by saline diuretic infusions.

**Tumours Complicating Pregnancy and Labour.**—Dr. Archibald Donald,[4] in an instructive paper on "Fibroid Tumours Complicating Pregnancy and Labour," sums up the risks during pregnancy as follows :—(1) Rapid increase in size of the tumour, causing severe pain and great distress ; (2) incarceration of the tumour in the pelvis ; (3) serious pressure on the bladder ; (4) degeneration of the tumour through diminished nutrition ; (5) excessive rotation of the pregnant uterus ; (6) abortion or premature labour. Abortion or labour might be complicated (1) by obstruction of the natural passages ; (2) by malpresentations ; (3) by retention of the placenta or membranes ; and (4) by extrusion of the tumour during labour. During the puerperium the presence of a uterine fibroid renders septic infection more likely. With regard to the treatment of these cases he recognises two main groups : (1) those in which the pregnancy is allowed to take its course until full term, or until the child is viable ; and (2) those in which it is necessary to interfere in the earlier months. In the majority of cases Dr. Donald thinks it is better to leave matters alone until term. If the tumour is located in the pelvis the treatment in each case must be settled by a careful examination. Cæsarian section is preferable to delivery by the natural passages if the latter involves the employment of much force. As regards treatment in the earlier months he discusses it under three headings : (1) induction of abortion ; (2) hysterectomy ; and (3) myomectomy. The induction of abortion ought, in Dr. Donald's opinion, to be abandoned, owing to the difficulty in emptying the uterus, and the liability of hæmorrhage and sepsis. He recommends that subperitoneal pedunculated fibroids should in all cases be removed during pregnancy, as there is considerable risk of torsion of the pedicle occurring during pregnancy and labour, while the risk of their removal is scarcely greater than that of ovariotomy. When the tumour is more sessile and operation is required he thinks it best to enucleate the tumour and stitch up the gap in the uterine wall. If, however, the raw surface is too extensive or the hæmorrhage difficult to control, the operation ought to be terminated by hysterectomy. J. Duncan Emmet[5] thinks that when pregnancy is complicated with the presence of a fibroid myomectomy is the best procedure. He reports a case where he operated early in the fourth month of pregnancy, and removed nine myomata. Labour took place at term, the placenta and membranes came away spontaneously in the course of half an hour and the uterus contracted firmly. The child was a healthy female, and as well as the mother was in good health six months after the operation.

**Uncontrollable Vomiting of Pregnancy.**—There is still considerable difference of opinion as to the cause of this troublesome and often dangerous complication of pregnancy. Some attribute it to inflammatory conditions of the cervix, uterine deviation, and tumours in the neighbourhood of the uterus ; others to a modification of the blood produced by the retention of toxic products. Professor Cristeanu[6] believes that a close relation exists between the arthritic diathesis and the vomiting. He maintains that gestation profoundly modifies the organism of the woman, and thus awakens the arthritic diathesis, diminishing on the one hand the disassimilation of the products of combustion, and retarding their elimination, with the consequence that toxic substances are accumulated in the organism. The necessity for the organism to eliminate these products probably by the gastric and buccal mucous membranes, leads as a natural consequence to irritation of these membranes, which react in their turn and produce the nausea and vomiting. He quotes several cases of his own which seem to support this theory. L. Lapeyre[7] reports a case of hyperemesis in a secundi para, who also had an ovarian cyst. Notwithstanding the removal of the cyst the vomiting continued, and the induction of abortion was rendered necessary, thus showing that the vomiting was due to the pregnancy alone, and not in any way connected with the presence of the cyst.

**Puerperal Infection.**—Dr. Arnold Lea[8] in an interesting paper on this subject points out that a more accurate knowledge of the bacterial invasion of puerperal infection is essential if further progress is to be made in the treatment. The uterine lochia from cases running a normal course are in the early days of the puerperium free from bacteria, though after the sixth or eighth day organisms are often present. In cases of suspected puerperal infection some of the uterine lochia should be obtained free from contamination with the vaginal discharge. Dr. Lea examined bacteriologically eighteen cases of puerperal infection, and found streptococcic infection in ten cases, staphylococcic infection in five cases, and infection with bacillus coli commune in one case. Anærobic bacteria were not investigated in every case, but in five cases they were found to be present. Streptococcic infection is by far the most common form, and presents enormous differences in virulence. The extraordinary differences in the results of streptococcic infection must be attributed to variations (1) in the virulence and type of the organism ; (2) in the resisting power of the individual. In pure streptococcic infection there is often no offensive odour of the lochia, which may be very scanty, or in some cases abundant and sanious. The treatment of puerperal infection must be mainly directed by the clinical symptoms, but bacteriological examination is also of value. If the uterine lochia are sterile, no intrauterine treatment is required. If the streptococcus is present, an attempt should be made to disinfect the uterus by curettage and other local measures. If bacteria of putrefaction are present, exploration of the uterus and removal of decomposing products is essential. The antistreptococcic serum should be given in cases of streptococcic infection. Albert[9] reports three interesting cases in which latent infection of the endometrium in pregnancy caused puerperal septic infection.

[1] Lancet, June 29, 1901. [2] Lancet, June 29, 1901. [3] Lancet, June 15, 1901. [4] Lancet, June 15, 1901. [5] Brit. Med. Jour., July 27, 1901. [6] Med. Press, Sept. 18, 1901. [7] Brit. Med. Jour., May 18, 1901. [8] Quar. Med. Jour., August, 1901. [9] Amer. Jour. of Med. Sci., September, 1901.

